# Cost–utility analysis of adapted problem adaptation therapy for depression in mild-to-moderate dementia caused by Alzheimer's disease: PATHFINDER randomised controlled trial

**DOI:** 10.1192/bjo.2024.775

**Published:** 2024-10-25

**Authors:** Monica Panca, Robert Howard, Elizabeth Cort, Charlotte Rawlinson, Rebecca L. Gould, Martin Wiegand, Anne Marie Downey, Sube Banerjee, Chris Fox, Rowan Harwood, Gill Livingston, Esme Moniz-Cook, Gregor Russell, Alan Thomas, Philip Wilkinson, Nick Freemantle, Rachael Maree Hunter

**Affiliations:** Comprehensive Clinical Trials Unit, University College London, UK; Division of Psychiatry, University College London, UK; Department of Statistical Science, University College London, UK; Priment Clinical Trials Unit, University College London, UK; Faculty of Medicine and Health Sciences, University of Nottingham, UK; College House, University of Exeter, UK; School of Health Sciences, University of Nottingham, UK; Faculty of Health and Social Care, University of Hull, UK; Bradford District Care NHS Foundation Trust, Shipley, UK; Campus for Ageing and Vitality, University of Newcastle, UK; Department of Psychiatry, University of Oxford, Warneford Hospital, Oxford, UK

**Keywords:** Dementia, depression, cost-effectiveness, cost-utility, quality-adjusted life years

## Abstract

**Background:**

Depression is common in people with dementia, and negatively affects quality of life.

**Aims:**

This paper aims to evaluate the cost-effectiveness of an intervention for depression in mild and moderate dementia caused by Alzheimer's disease over 12 months (PATHFINDER trial), from both the health and social care and societal perspectives.

**Method:**

A total of 336 participants were randomised to receive the adapted PATH intervention in addition to treatment as usual (TAU) (*n* = 168) or TAU alone (*n* = 168). Health and social care resource use were collected with the Client Service Receipt Inventory and health-related quality-of-life data with the EQ-5D-5L instrument at baseline and 3-, 6- and 12-month follow-up points. Principal analysis comprised quality-adjusted life-years (QALYs) calculated from the participant responses to the EQ-5D-5L instrument.

**Results:**

The mean cost of the adapted PATH intervention was estimated at £1141 per PATHFINDER participant. From a health and social care perspective, the mean difference in costs between the adapted PATH and control arm at 12 months was −£74 (95% CI −£1942 to £1793), and from the societal perspective was −£671 (95% CI −£9144 to £7801). The mean difference in QALYs was 0.027 (95% CI −0.004 to 0.059). At £20 000 per QALY gained threshold, there were 74 and 68% probabilities of adapted PATH being cost-effective from the health and social care and societal perspective, respectively.

**Conclusions:**

The addition of the adapted PATH intervention to TAU for people with dementia and depression generated cost savings alongside a higher quality of life compared with TAU alone; however, the improvements in costs and QALYs were not statistically significant.

Dementia is a group of symptoms associated with a decline in memory that affects daily living. Alzheimer's disease is a degenerative brain disease caused by complex brain changes following cell damage, and leads to gradual worsening of dementia symptoms over time.^1^

The number of people with dementia is rising with an ageing population. In England, it is estimated that the cost of long-term care for older people with dementia will increase from £5.4 billion in 2002 to £16.7 billion in 2031.^2^

Depression is common in people with dementia, with 20% experiencing a major depressive disorder.^3^ Depression in dementia reduces quality of life,^4^ increases mortality,^5^ increases the probability of care home admission^6^ and increases carer burden.^7^ Treating depression in dementia should be seen as a priority, with the potential to improve quality of life and level of function; it might also influence costs, as depression imposes a considerable disability burden.^8^

Several studies have been conducted to assess the impact of antidepressants^9,10^ and psychological therapies^11^ on depression in people with dementia, but they have shown little or no benefit. An economic evaluation conducted alongside the HTA-SADD (Sertraline or mirtazapine for depression in dementia) trial^12^ showed that mirtazapine and sertraline were not cost-effective for treating (reducing) depression in dementia, but when costing included the impact on unpaid carers and quality of life was the economic outcome, mirtazapine was cost-effective.

Problem-solving therapy studies have been conducted aiming to reduce the negative impact of behavioural functional limitations through a range of emotional regulation techniques to increase positive emotions (e.g. pleasure) and decrease negative emotions (e.g. sadness, anxiety). The Problem Adaption Therapy (PATH) study^13^ was based on problem-solving therapy among depressed and mildly cognitively impaired older people, and was reported to improve depression symptoms. However, the PATH study focused on short-term outcomes and therefore has not demonstrated medium- or longer-term benefits. It is believed that the use of booster sessions beyond the initial treatment period provides the potential for extending the benefits of PATH (PATH-MCI; Clinicaltrial.gov identifier NCT03043573).

## Study aims

The Problem Adaptation Therapy for Individuals with Mild to Moderate Dementia and Depression (PATHFINDER) randomised controlled trial was designed to investigate the clinical effectiveness and cost-effectiveness of an adapted PATH intervention for depression in mild-to-moderate dementia caused by Alzheimer's disease. The economic evaluation reported here, conducted alongside the PATHFINDER trial in 24 centres in England and Wales, assessed the cost-effectiveness of the adapted PATH intervention.

## Method

### Trial design and population

Participants (*n* = 336) were recruited from National Health Service (NHS) memory services, community mental health services for older people, primary care and third-sector services for people with mild or moderate dementia owing to Alzheimer's disease, who scored >7 on the Cornell Scale for Depression in Dementia (CSDD),^14^ were living at home, and were not diagnosed with schizophrenia or bipolar disorder or expressing suicidal ideation. Participating patients and their caregivers gave written informed consent for inclusion.

The PATH intervention^13^ was adapted^15^ for the current study to make it deliverable in the NHS and UK cultural context, using a person-centred qualitative approach by members of the original PATH team and PATHFINDER investigators, specifically for use with people with the full range of cognitive impairment in moderate dementia and major depression. Participants were randomised with a 1:1 allocation to receive adapted PATH in addition to usual multidisciplinary care (the intervention arm, ‘adapted PATH’) or usual multidisciplinary care alone (the control arm, treatment as usual (TAU)), stratified by antidepressant use. Randomisation was performed with a web-based internet randomisation service provided by Sealed Envelope^TM^ (https://www.sealedenvelope.com/). Participants randomised to the intervention arm were offered up to eight initial manualised 1 h PATH sessions over 12 weeks, delivered by a trained and supervised therapist. Participants were accompanied by caregivers who were involved as co-therapists. The therapy comprised two assessment sessions, five sessions focused on problem-solving with PATH tools, and one review session supplemented by 1 h booster sessions at 6 and 9 months to review key strategies used in PATH.

The Wales Research Ethics Committee 4 Wrexham granted ethical approval on 14 June 2018 (Integrated Research Application System identifier 238724). The trial was preregistered with the ISRCTN Registry on 31 May 2018 (identifier ISRCTN11185706) and the trial protocol is available at https://fundingawards.nihr.ac.uk/award/16/155/01.

### Overview

The economic evaluation was conducted with individual participant cost and effect data collected alongside the PATHFINDER trial, and was performed from both a health and social care (NHS and Personal Social Services (PSS)) perspective and a broader societal perspective, including out-of-pocket and informal care costs, besides the health and social care. The analysis was conducted on an intention-to-treat basis and all costs are reported in 2021–2022 UK pounds (£), adjusted for inflation where necessary, using the NHS Cost Inflation Index.^16^ The time horizon of the analysis was 12 months, with assessments of costs and outcomes at the following points: baseline and 3-month, 6-month (all asking for previous 3 months) and 12-month follow-up (asking for previous 6 months). Discounting was not applied given that the duration of follow-up did not exceed 12 months. The report of this health economic evaluation followed the Consolidated Health Economics Evaluation Reporting Standards (CHEERS) guidance.^17^

### Identification, measurement and valuation of health and social care resource use

#### Set-up costs and delivery of the intervention

A micro-costing approach (a cost estimation method that involves direct enumeration of the cost of each resource required^18^) was adopted to estimate the additional resource use and costs associated with the adapted PATH intervention. Study records of the number of therapists attending training sessions were used to track resources used in the delivery of the training programmes, including trainee and trainer time (and preparation time), travel costs, attendance incentives and course materials, to calculate the fixed cost of training. For the delivery of the intervention, the number of sessions delivered and the time each therapist spent with a participant were recorded and any materials provided to participants. Unit costs for therapists to train for and deliver the intervention were based on the most recently available national estimates.^16^ Actual expenses incurred for training materials, refreshments and therapists travel were also recorded.

#### Health and social care utilisation and personal expenditure on healthcare

NHS healthcare, community and social services and participant personal resource use during the 12 months of follow-up were captured with a resource-use questionnaire adapted from the Client Service Receipt Inventory (CSRI).^19^ This is a measure of service utilisation used to calculate participant and caregiver costs, which focused on treatments for depression that participants accessed as well as other depression-related health and social care service use. The questionnaire included in-patients stays, out-patient attendances, day hospital treatment and contact with community-based professionals.

Adaptations or changes to participants’ home including memory aids and alarms were also recorded. The questionnaire was also used to record other forms of psychological therapies received outside of the study and unpaid support provided by friends and family. In addition, we also collected information about caring activities of unpaid caregivers for the participant. Respondents were asked to estimate the hours of unpaid care and support from family/friends. Opportunity costs were attached to these hours using the cost of lost employment. Medication data were obtained from the trial medication log.

Health and social care resource use were costed using unit costs from the most recent unit costs of health and social care published by the Personal Social Services Research Unit^16^ and NHS reference costs^20^ (Supplementary Table 1 available at https://doi.org/10.1192/bjo.2024.775) supplemented by micro-costing or local estimates if necessary. The costs of medications were estimated from the British National Formulary.^21^ Informal caregivers were costed at the rate of paid caregivers based on the assumption that in the absence of an informal caregiver a paid carer would be required to undertake the same role.^22^ An hour of unpaid care was costed as the median hourly wage of a home care worker. The health and social care cost was derived by combining primary, community services and medication, and the societal cost was derived by combining health and social care cost with out-of-pocket expenses and the (opportunity) cost of time spent caregiving by family and friends. The cost of each resource item was calculated by multiplying the number of resource units used by the unit cost. The total cost for each participant was then estimated as the sum of the cost of resource use items consumed.

### Identification, measurement and valuation of outcomes

The primary health outcome measure used in the economic evaluation was quality-adjusted life-years (QALYs). The QALY is the reference case outcome recommended by the National Institute for Health and Care Excellence (NICE) for use in economic evaluations,^23^ and allows for the comparison of the economic case for interventions across all health-related interventions, regardless of the specific health condition being assessed.

Primary analysis included QALYs calculated from participant responses to the EQ-5D five-level (EQ-5D-5L)^24^ instrument. The EQ-5D-5L is a generic health-related measure of quality of life that contains five domains: mobility, self-care, usual activities, pain/discomfort and anxiety/depression; each domain has five levels. Each of the five items is rated on a five-point scale, from no problem to extreme problems. The self-completed questionnaire captured participant perspective on health status. The crosswalk algorithm,^25^ which maps the EQ-5D-5L values sets to the currently available three-level version of the EQ-5D-3L, was used for the primary analysis.^23^

Supporting analyses included QALYs calculated from responses to the Dementia Quality of Life questionnaire (DEMQOL), a dementia-specific preference-based measure of quality of life,^26^ to generate utility values for every health state defined by the health-state classification systems derived from DEMQOL (28 items) and DEMQOL-Proxy (31 items).^27^ The interviewer-administered questionnaires captured self and informant (carer) reports of the health-related quality of life (HRQoL) covering feelings, memory and everyday life of the person with dementia. Each of the items is rated on a Likert scale (a lot/quite a bit/a little/not at all). The DEMQOL instruments can be used alongside a generic preference-based measure such as the EQ-5D instruments in studies of interventions in dementia.^27^

EQ-5D and DEMQOL utility scores enabled the calculation of QALYs with the area under the curve method.^28^

### Statistical analysis

All analyses were conducted with the intention-to-treat principle, comparing the two arms as randomised and including all participants in the analysis. Analyses conformed to accepted economic evaluation methods.^23^

Mean incremental costs and QALYs were analysed with mixed-effects logistic regressions, adjusting for baseline values, treatment allocation and baseline use of antidepressant medication (stratification factor) as fixed effects, with sites as random effects. A bias-corrected and accelerated bootstrap method will be used to calculate 95% confidence intervals.

Estimates of bootstrapped mean cost and effectiveness were used to estimate an incremental cost-effectiveness ratio (ICER). The ICER for each replication was calculated by dividing the difference in total costs (incremental cost) by the difference in total health outcome (incremental effect), to provide a ratio of extra cost per extra unit of health effect.

Uncertainty around cost-effectiveness outcomes was modelled by plotting bootstrapped results for incremental costs and outcomes on cost-effectiveness planes (CEPs).^29^ These were used to inform the cost-effectiveness acceptability curves (CEACs), where the probability that the adapted PATH intervention was cost-effective was plotted against a range of cost-effectiveness thresholds.^30^

### Missing data

To account for missing data, we performed multiple imputation by chained equations^31^ under the missing-at-random (MAR) assumption, to impute missing values for resource use costs and utility values. The analysis was performed in Stata version 16^32^ and in total, 46 data-sets were imputed, with site, gender, age, use of antidepressants and baseline depression level included in the imputation model as baseline predictors of missingness. Participants were excluded from the multiple imputation analysis if they had no EQ-5D-5L (or DEMQOL/DEMQOL-Proxy in supporting analyses) or CSRI entries at baseline. Mixed-effects logistic regression was used to calculate baseline-adjusted differences in costs and outcomes, and subsequently, incremental costs and incremental outcomes. We ran the analytic model within each of the imputed data-sets, using Rubin's rules to control for the variability between imputations. Bootstrapping was used only in conjunction with multiple imputation to display uncertainty in the form of CEPs and CEACs.

### Sensitivity analysis

A sensitivity analysis was conducted considering the delivery of the intervention costs alone, excluding the costs of therapists training and consumables, as this would more closely reflect the cost of delivering adapted PATH as a routine service in practice.

The COVID-19 pandemic affected the trial from 26 March 2020, when lockdown rules were imposed in the UK. Although adapted PATH intervention sessions or research follow-up visits were not able to be delivered or conducted face to face, these were carried out via telephone or videoconference systems (e.g. Skype, Zoom). This approach attempted to minimise the loss of data. Cognitive function measures were not always possible to administer fully over the telephone, and in such cases, some items were necessarily missed. Researchers administered the outcome measures as completely as possible, bearing in mind the limitations of not being able to meet face to face. If the participant with dementia became less able to use the telephone or engage with the video call, then the therapist completed the questionnaires with the caregiver. This may have affected the cost of the intervention. COVID-19 may have had an impact on participants’ and carers’ mental health and HRQoL during this period. Therefore, we performed a sensitivity analysis to capture the effect of COVID-19 restrictions on costs and outcomes. A binary variable was created if data collection at each time point was before (0) or after (1) restrictions were imposed (26 March 2020), and was included in bias-corrected and accelerated bootstrapped regressions for costs and outcomes.

When the health and social care resource use data were collected at 3- and 6-month follow-up points during the COVID-19 restrictions, researchers were asked to omit specific questions (all of which were asking if participants had been seen by a psychologist or had psychological therapy) to maintain blindness to intervention arm. However, this information was collected retrospectively by checking care notes or asking the clinical team/general practitioner/memory service/psychology team. Data were considered recorded at 3 and 6 months because these data were collected from each participant at these time points. Data not collected at these time points were considered missing at those time points for the purpose of the statistical analysis. A major concern was that the chance of missing data may have been directly linked to the unobserved value itself. However, the extent to which this has occurred could not be established from the data. Therefore, to evaluate the uncertainty around this assumption and avoid bias, we conducted a sensitivity analysis under the missing-not-at-random (MNAR) assumption, to evaluate the robustness of the results.^33^ MNAR occurs when missingness is dependent on unobserved factors, and this may introduce bias if, for example, individuals are more likely to have missing data depending on if they have good or bad outcomes. However, to evaluate the uncertainty around this assumption and avoid bias, we have explored how the results may change if we assumed the data were MNAR. We used the recommended scenario analysis,^33^ modifying the MAR-imputed data to reflect plausible MNAR scenarios considered departures from the MAR assumption for the cost end-point. Therefore, we evaluated the percentage of participants who had missing data at the follow-up points where the psychological therapy attendances data were not collected (3 or 6 months) during the COVID-19 restrictions, but who had entries at the next follow-up point (6 or 12 months), and used them to modify the MAR-imputed data.

If the results of the sensitivity analyses and the original analysis are inconsistent, the impact of missing data on the analysis should be considered a limitation and would restrict the strength of the conclusions drawn from the data.

## Results

A total of 1238 patients were screened for eligibility, of whom 363 met the inclusion criteria; 336 participants were randomised between 24 September 2019 and 22 January 2022, with 168 randomised to the adapted PATH arm and 168 randomised to the TAU arm. The median age of the participants was 78 years in the adapted PATH arm and 76 years in the TAU arm. Participants were predominantly female (60%), with a mean (s.d.) baseline CSDD score of 13 (s.d. 3.75) in the adapted PATH arm and 12.8 (s.d. 3.75) in the TAU arm. Further details can be found in Supplementary Table 2 and in the main trial paper.^34^ At 12 months, complete cost and utility data were available for 109 (65%) participants in the adapted PATH arm and 106 (63%) in the TAU arm. The Consolidated Standards of Reporting Trials (CONSORT) diagram is reported in Supplementary Fig. 1.

### Set up and delivery of the intervention estimated costs

Two Band 9 and one Band 7 academic professional staff (research team) delivered training for 21 days, in 7 h face-to-face sessions (or 6 h remotely during COVID-19 restrictions) plus 2 h booster sessions, for 187 individuals (NHS Bands 4–7 therapists and NHS Band 8 supervisors). Other costs included training materials and the therapy manual.

Eighty-two therapists delivered 740 h of adapted PATH sessions (mean duration: 1 h per session) with fortnightly supervision (two to three therapists per session) provided locally throughout the intervention delivery period by supervisors (clinical psychologists). There were 51 sessions delivered face to face, 41 hybrid sessions (face to face and remote) and 37 remote-only sessions. The cost of the intervention included only the costs of training the 82 therapists and 24 supervisors who were involved in delivering the intervention.

Total cost of the adapted PATH intervention was estimated at £212 268. Given that 168 participants in the adapted PATH arm received the intervention, this translated to a conservative estimate of £1141 per PATHFINDER participant in the intervention arm, given that therapists are likely to deliver the intervention to a greater number of patients than this if introduced into routine practice (Table 1). A total of 61% of the total costs related to the therapists’/supervisors’ time to deliver the intervention.

**Table 1:** Estimated cost of the adapted Problem Adaption Therapy study intervention

Description	Total cost (£)
Training costs	
Band 9 (*n* = 2) and Band 7 (*n* = 1) delivered training for 21 days, 7 h face to face/6 h remotely (+ 2 h refresher) training time for 187 individuals	27 759
Face to face	
Supervisor	
Band 8 trainee training session time costs	13 140
Therapist	
Band 4 trainee training session time costs	2142
Band 5 trainee training session time costs	738
Band 6 trainee training session time costs	12 402
Band 7 trainee training session time costs	954
Specialty trainee ST6	954
Travel costs	2602
Remote	
Supervisor	
Band 8 trainee training session time costs	2336
Therapist	
Band 4 trainee training session time costs	2448
Band 5 trainee training session time costs	328
Band 6 trainee training session time costs	13 144
Specialty trainee ST6	424
Specialty trainee ST7	424
Total training costs	79 795
Session delivery	
Sessions delivered (mean time per session 1 h) (Band 4–7)	36 149
Supervision fortnightly (mean time per session 1 h) one supervisor (Band 8) and two to three therapists per session	93 539
Total session delivery costs	129 688
Other costs	
Training sessions	
Notebooks	451
Folders	121
Catering	485
Film	200
Printing	396
Postage of therapy worksheets	612
Intervention delivery	
Manual for therapists	275
Intervention sheets for participants	246
Total other costs	2785
Total cost	212 268
Cost per participant in intervention arm	1141

### Health and social care utilisation and personal expenditure on healthcare

The CSRI listed the health and social services with which the participants could have contacts between follow-up points. In addition, an open question allowed participants to indicate other health and social services they had contact with. Such contacts made by study participants were recorded, and our results showed that participants in the adapted PATH arm reported statistically significantly fewer contacts with other community practitioners (e.g. older people services, community dementia support nurse) over the 12 months of the trial (baseline-adjusted difference: −0.63; 95% CI −1.24 to −0.02). There were no other statistically significant differences between the trial arms (Supplementary Table 3).

Supplementary Table 4 reports the baseline-adjusted mean difference in cost of health and social service use at 12 months for both the intervention and control arms. There were no statistically significant differences in healthcare service costs between the trial arms. For participants in the adapted PATH arm, costs were higher for state-funded help (baseline-adjusted difference: £311; 95% CI −£572 to £1195), unpaid help (baseline-adjusted difference: £163; 95% CI −£11 to £336) and family help (baseline-adjusted difference: £318; 95% CI −£28 to £664), whereas for participants in the TAU arm, costs were higher for privately funded help (baseline-adjusted difference: £550; 95% CI −£2883 to £3983) and overnight in-patient hospital stay (baseline-adjusted difference: £1542; 95% CI −£233 to £3317). Excluding the cost of the adapted PATH intervention, the baseline-adjusted difference in total costs for health and social care at 12 months between trial arms (adapted PATH versus TAU) was −£1342 (95% CI −£3539 to £855) and the baseline-adjusted difference in the societal total cost at 12 months between trial arms was −£270 (95% CI −£8304 to £7764).

### Outcomes

The primary health economic outcome was QALYs gained over 12 months, estimated using the EQ-5D-5L. Mean EQ-5D-5L scores in each arm at baseline and all follow-up points, and the baseline-adjusted QALYs are reported in Supplementary Table 5. The results showed that mean EQ-5D-5L scores improved over the study period for both trial arms. There was a statistically significant baseline-adjusted difference of 0.047 (95% CI 0.011–0.083; *P* = 0.011) in QALYs at 12 months in favour of the adapted PATH arm.

Supporting analyses were conducted with QALYs constructed using utility values based on responses to DEMQOL and DEMQOL-Proxy questionnaires (Supplementary Table 5). The results showed that the mean DEMQOL/DEMQOL-Proxy scores also improved over the study period for both trial arms. There were positive QALY estimates, but the baseline-adjusted differences between arms were not statistically significant for QALYs derived from the DEMQOL (baseline-adjusted difference: 0.009; 95% CI −0.015 to 0.033) and QALYs derived from the DEMQOL-Proxy (baseline-adjusted difference: 0.012; 95% CI −0.007 to 0.030).

#### Cost–utility analysis

The primary economic evaluation was a within-trial cost–utility analysis over 12 months, from the NHS/PSS cost perspective. Following multiple imputation, the mean difference in costs between the adapted PATH and control arm at 12 months was −£74 (95% CI −£1942 to £1793) and the mean difference in QALYs was 0.027 (95% CI −0.004 to 0.059) (Table 2).

**Table 2:** Mean incremental costs, quality-adjusted life-years, incremental cost-effectiveness ratios and probabilities of the adapted Problem Adaption Therapy study intervention being cost-effective at £20 000 and 30 000 per quality-adjusted life-year gained value thresholds (multiple imputation)

	Incremental cost	Incremental benefit	ICER	Probability of cost-effectiveness at £20 000 per QALY gained	Probability of cost-effectiveness at £30 000 per QALY gained
NHS/PSS perspective					
QALYs from EQ-5D-5L	−£74 (−£1942 to £1793)	0.027 (−0.004 to 0.059)	Dominant	74%	80%
QALYs from DEMQOL	−£74 (−£1942 to £1793)	0.009 (−0.009 to 0.292)	Dominant	64%	66%
QALYs from DEMQOL-Proxy	−£74 (−£1942 to £1793)	0.011 (−0.006 to 0.027)	Dominant	66%	68%
Societal perspective					
QALYs from EQ-5D-5L	−£671 (−£9144 to £7801)	0.027 (−0.004 to 0.059)	Dominant	68%	70%
QALYs from DEMQOL	−£671 (−£9144 to £7801)	0.009 (−0.009 to 0.292)	Dominant	65%	65%
QALYs from DEMQOL-Proxy	−£671 (−£9144 to £7801)	0.011 (−0.006 to 0.027)	Dominant	65%	66%

ICER, incremental cost-effectiveness ratio; QALY, quality-adjusted life-year; NHS/PSS, National Health Service/Personal Social; EQ-5D-5L, EuroQol-5D five level; DEMQOL, Dementia Quality of Life questionnaire.

**Fig. 1:**
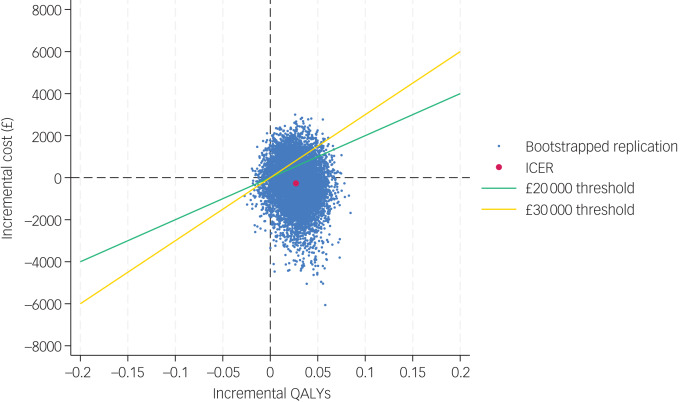
Cost-effectiveness plane of the adapted Problem Adaption Therapy study intervention compared with treatment as usual from a National Health Service/Personal Social Services cost perspective at 12 months, using QALYs derived from the EQ-5D-5L. ICER, incremental cost-effectiveness ratio; QALY, quality-adjusted life-year.

**Fig. 2:**
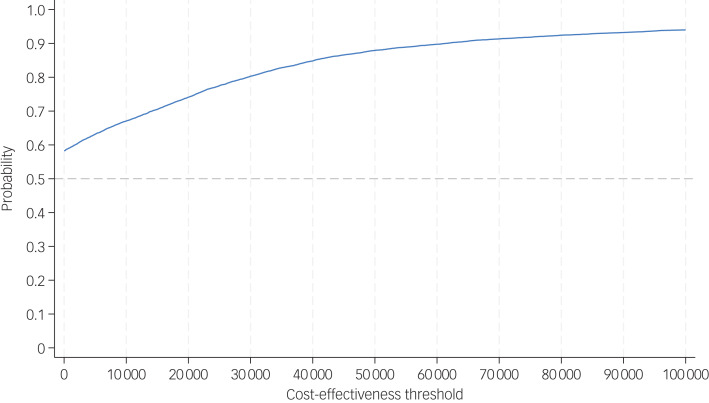
Cost-effectiveness acceptability curve of adapted Problem Adaption Therapy study intervention compared with treatment as usual from National Health Service/Personal Social cost perspective at 12 months, using quality-adjusted life-years derived from the EQ-5D-5L.

The CEP and CEAC were constructed with the two-step-bootstrapping following multiple imputation, and are shown in Figs 1 and 2. From the NHS/PSS perspective, the probabilities that the adapted PATH intervention was cost-effective compared with TAU were 74 and 80% at cost-effectiveness thresholds of £20 000 and £30 000 per QALY gained.

Results from the broader societal perspective, including out-of-pocket and informal care costs as well as health and social care costs, showed that, following multiple imputation, the mean difference in costs between adapted PATH and TAU arms at 12 months was −£671 (95% CI −£9144 to £7801). From the societal perspective, the probabilities that the adapted PATH intervention was cost-effective compared with TAU were 68 and 70% at cost-effectiveness thresholds of £20 000 and £30 000 per QALY gained (Table 2).

In the supporting analyses, from the NHS/PSS perspective, the mean difference in QALYs between adapted PATH and TAU arms at 12 months was 0.009 (95% CI −0.009 to 0.292) when QALYs were derived from the DEMQOL, and 0.011 (95% CI −0.006 to 0.027) when QALYs were derived from the DEMQOL-Proxy (Table 2). The probabilities that the adapted PATH intervention was cost-effective compared with TAU in the supporting analyses are presented in Table 2.

### Sensitivity analyses

The total cost of delivering the adapted PATH intervention alone (excluding costs of therapists training and consumables) was estimated at £129 688. This translates in an estimate of £772 per PATHFINDER participant in the intervention arm. From the NHS/PSS cost perspective, following multiple imputation, the mean difference in costs between the adapted PATH and control arm at 12 months was −£443 (95% CI −£2311 to £1424). The probability that the adapted PATH intervention was cost-effective compared with TAU when only the delivery of the intervention cost was included was 84 and 89% at cost-effectiveness thresholds of £20 000 and £30 000 per QALY gained, respectively (Supplementary Fig. 2).

Recruitment and follow-up for the trial occurred in part during the COVID-19 pandemic, which may have had an impact on the ability to access health and social care services and some responses to patient-reported outcomes. However, our sensitivity analysis found no impact of COVID-19 restrictions on the results, and the probability of the adapted PATH intervention being cost-effective remained the same as in the main analysis. The probability that the adapted PATH intervention was cost-effective compared with TAU was 65 and 77% at cost-effectiveness thresholds of £20 000 and £30 000 per QALY gained, respectively (Supplementary Fig. 3).

More participants in the TAU arm had missing data for psychological therapies (38% at 3 months and 47% at 6 months), whereas only 22% of participants in the adapted PATH arm had missing data at 6 months. Therefore, using the approach described by Leurent et al,^33^ we built three scenarios around these findings: scenario 1, no departure from MAR; scenario 2, a 0.6 weight applied for the TAU arm and scenario 3, a 0.5 weight applied for the TAU arm and 0.8 weight applied for the adapted PATH arm. The results of the three scenarios analyses conducted under the assumption that data was MNAR are presented on a CEP (Supplementary Fig. 4) and the probability of cost-effectiveness on the CEAC (Supplementary Fig. 5). Our findings showed that departure from the MAR assumption for costs could importantly affect the conclusions, particularly if the missing costs were assumed only in the TAU arm (48% probability of adapted PATH to be cost-effective compared with TAU at a threshold value of £20 000 per QALY gained).

## Discussion

Our results showed that at 12 months, the adapted PATH intervention dominated TAU in that it cost £74 (95% CI −£1942 to £1793) less than TAU from the NHS/PSS perspective with a positive mean point estimate for QALYs obtained from EQ-5D-5L (0.027; 95% CI −0.004 to 0.059). There was a reasonably high probability that the adapted PATH intervention is cost-effective (74%) for people with dementia suffering from depression when patient quality of life is taken in account. Analyses conducted from a broader societal perspective produced similar findings, but there is a 68% probability that the adapted PATH intervention is cost-effective at a cost-effectiveness threshold of £20 000 per QALY gained.

Previous studies of dementia^35^ and depression^12,36^ have reported similar results to ours in the sense that the intervention showed a cost-effectiveness advantage when using the QALY as the outcome measure despite the fact that there was no difference on the primary measure.

### Strengths and limitations of the study

A key strength of this study is that it included a full health economic evaluation compliant with NICE health technology assessment guidelines.^23^

The PATHFINDER trial (main analysis) found that the adapted PATH intervention did not reduce symptoms of depression compared with TAU on the CSDD scale at 6 months, and concluded that it was not possible to recommend the addition of the adapted PATH intervention to TAU for the reduction of depression in people with dementia.^34^ Despite the lack of clinical effectiveness, the economic evaluation showed that the adapted PATH intervention may have positive effects on quality of life in this population. However, the results should be interpreted cautiously, as confidence intervals were wide.

Although there is an ongoing debate about the appropriateness of using the EQ-5D instrument in people with dementia, this is the NICE-preferred approach for generating utility data across interventions and conditions.^23^ Also, there is increasing evidence of the validity and responsiveness of the EQ-5D-5L in depression.^37^ We also used the dementia specific preference-based measure DEMQOL to generate utility data. Responses to the DEMQOL/DEMQOL-Proxy also produced positive baseline-adjusted differences in QALYs at 12 months; however, they were not statistically significant.

As with other studies based on self-reported outcome measures or proxy-reported resource use questionnaires, our study had a considerable proportion of participants with missing HRQoL or cost data. There were some incomplete data from the CSRI, which was to be expected given the size and spread of the sample and the comprehensive nature of the service use data collection exercise. Also, COVID-19 restrictions meant that there was a risk of unblinding the researchers, therefore some of the data was not collected at the 3- and 6-month follow-up time points. TAU could not be delivered in the conventional way, as most of the services available in local areas were interrupted or completely suspended during the period of COVID-19 restrictions (March 2020 to January 2022) and beyond. Therefore, participants recruited in this study during this time had little to no help available in their local communities, with possible impact on the cost. To reduce the amount of missing data, researchers collected these data from alternative sources at the end of the study. Missing responses were therefore assigned a value by imputation to make efficient use of the data provided. We have used a widely recommended approach, multiple imputation, for handling missing data in cost-effectiveness analysis.^31^ Although we have attempted to address this by multiple imputation (MAR and MNAR), the approach will not have completely overcome the potential bias implicit in incomplete follow-up data.

#### Implications

As policy makers focus on health and social care costs, the findings reported here suggest that using an intervention similar to ours for treating depression in people with dementia could be cost-effective if a broader focus on health outcomes is adopted. The PATHFINDER trial (main analysis) found that small improvements with adapted PATH were seen at 3 months, but not at 6 months, on the CSDD.^34^ Therefore, such interventions could be relatively cheap if they were to be implemented within the NHS, as therapy providers could be trained locally and could be cost-saving in the long-term if the short-term improvements last. Therefore, much depends on the way programmes are implemented outside clinical trials and the extent to which people with dementia attend and experience long-term benefits.

Evidence about the cost-effectiveness of adapted PATH can help decision makers make more efficient use of scarce resources. Decisions about health resource allocation should be based on the relative benefits and costs of interventions, although these cannot be the sole criteria used. Considering the study findings that the adapted PATH intervention did not reduce depression in people with dementia compared with TAU at 6-month follow-up,^34^ it is essential that policy makers consider whether adapted PATH could be delivered as an intervention for people with dementia and poor quality of life. There is potential in the adapted PATH intervention, as our results showed improvement in quality of life despite no reduction in depression. Therefore, these findings need further development and re-evaluation (with more emphasis on quality of life). Also, in light of the COVID-19 disruption, this has undoubtedly distorted costs (services suspended or not available, family members not going out to work, remote intervention delivery) and may have distorted outcomes (although presumably affecting both arms, but there may have been statistical interaction if adapted PATH differentially helped with adaptation to COVID-19 restrictions).

With these limitations, further research could assess whether the adapted PATH intervention could be provided to a targeted population (e.g. different levels of cognitive impairment, different levels of HRQoL) or should examine longer treatment periods as well as potential benefits of combining adapted PATH with antidepressants, along with other components of comprehensive dementia care management.

## Supporting information

Panca et al. supplementary material Panca et al. supplementary material

## Data Availability

Data supporting the findings can be obtained on reasonable request from the corresponding author, M.P. Requests for access to the PATHFINDER data will be reviewed on an individual basis by the Chief Investigator.
