# Supplementary material for: Cost–utility analysis of adapted problem adaptation therapy for depression in mild-to-moderate dementia caused by Alzheimer's disease: PATHFINDER randomised controlled trial
Source: BJPsych Open. 2024 Oct 25;10(6):e189. doi: 10.1192/bjo.2024.775 (PMC11698147; doi:10.1192/bjo.2024.775)
Supplement: Panca et al. supplementary material [file S2056472424007750sup001.docx]

**Supplementary information**

**Table S1. Unit costs applied for valuation of health and social care resource use**

| **Resource category** | **Unit cost (2021/2022) (£)** | **Notes** | **Source** |
| --- | --- | --- | --- |
| **Primary care and community health** | | | |
| GP at clinic | £38 | Per surgery consultation lasting 9.22 minutes | PSSRU 2022 |
| GP by phone | £9 | Assumed 5 minutes phone consultation | PSSRU 2022 |
| GP at home | £236 | Annual inflator to uprate the costs to 2021/2022 values (The Personal Social Services (PSS) Pay & Prices Index) | PSSRU 2015 |
| Practice Nurse at clinic | £13 | Assumed 15 minutes consultation | PSSRU 2022 |
| Practice Nurse by phone | £9 | Assumed 5 minutes phone consultation | PSSRU 2022 |
| Practice Nurse at home | £22 | Assumed equivalent to a home visit of 25 minutes. | PSSRU 2022 |
| Community/ District Nurse at clinic | £22 | Assumed 30 minutes consultation; Annual inflator to uprate the costs to 2021/2022 values (The Personal Social Services (PSS) Pay & Prices Index) | PSSRU 2018 |
| Community/ District Nurse by phone | £7 | Assumed 10 minutes consultation | PSSRU 2022 |
| Community/ District Nurse at home | £69 | Annual inflator to uprate the costs to 2021/2022 values (The Personal Social Services (PSS) Pay & Prices Index) | PSSRU 2018 |
| CMHT Doctor at clinic | £72 | Assumed 30 minutes consultation | PSSRU 2022 |
| CMHT Doctor by phone | £24 | Assumed 10 minutes phone consultation | PSSRU 2022 |
| CMHT Doctor at home | £60 | Assumed equivalent to a home visit of 25 minutes | PSSRU 2022 |
| CMHT Occupational Therapist/ Social Worker at clinic | £25 | Assumed 30 minutes consultation; Annual and unit costs for community-based scientific and professional staff, Band 7 | PSSRU 2022 |
| CMHT Occupational Therapist/ Social Worker by phone | £8 | Assumed 10 minutes phone consultation | PSSRU 2022 |
| CMHT Occupational Therapist/ Social Worker at home | £21 | Assumed equivalent to a home visit of 25 minutes | PSSRU 2022 |
| CMHT Psychologist at clinic | £63 | Assumed 30 minutes consultation; Annual and unit costs for community-based scientific and professional staff, Band 8 | PSSRU 2022 |
| CMHT Psychologist by phone | £21 | Assumed 10 minutes phone consultation | PSSRU 2022 |
| CMHT Psychologist at home | £52 | Assumed equivalent to a home visit of 25 minutes | PSSRU 2022 |
| CMHT Assistant Practitioner at clinic | £21 | Assumed 30 minutes consultation | PSSRU 2022 |
| CMHT Assistant Practitioner by phone | £7 | Assumed 10 minutes phone consultation | PSSRU 2022 |
| CMHT Assistant Practitioner at home | £17 | Assumed equivalent to a home visit of 25 minutes | PSSRU 2022 |
| **Psychological therapy** | | | |
| Individual therapy | £40 | Per 50-minutes session | https://wpf.org.uk/therapy/fees/ |
| Group therapy | £30 | Per 1 hour session | https://wpf.org.uk/therapy/fees/ |
| **Emergency services** | | | |
| NHS direct or “Call 111” | £11.40 |  | Impact of NHS 111 Online on the  NHS 111 telephone service and urgent  care system: a mixed-methods study - https://www.ncbi.nlm.nih.gov/books/ NBK575180/pdf/Bookshelf_NBK575180.pdf |
| Emergency call (999) | £89.59 | Annual inflator to uprate the costs to 2021/2022 values (The Personal Social Services (PSS) Pay & Prices Index) | National Schedule of NHS Costs - Year 2020-21/Ambulance/ Hear & Treat |
| Paramedic only | £268.43 | Annual inflator to uprate the costs to 2021/2022 values (The Personal Social Services (PSS) Pay & Prices Index) | National Schedule of NHS Costs - Year 2020-21/Ambulance/ See & Treat |
| Paramedic and ambulance to hospital | £357.40 | Annual inflator to uprate the costs to 2021/2022 values (The Personal Social Services (PSS) Pay & Prices Index) | National Schedule of NHS Costs - Year 2020-21/Ambulance/ See & Convey |
| A&E attendance WITHOUT ambulance | £170 | Annual inflator to uprate the costs to 2021/2022 values (The Personal Social Services (PSS) Pay & Prices Index) | National Schedule of NHS Costs - Year 2020-21/ Total Outpatient Attendance / Emergency Medicine Service |
| **Overnight Inpatient stay** | | | |
| Unplanned (emergency) inpatient hospital stay for physical health reasons | £968 | Annual inflator to uprate the costs to 2021/2022 values (The Personal Social Services (PSS) Pay & Prices Index) | National Schedule of NHS Costs - Year 2020-21, Non Elective Inpatients (trim point floor of five days -https://www.england.nhs.uk/wp-content/uploads/2020/11/21-22NT_Annex-B-Guidance-on-currencies.pdf |
| Planned (elective) inpatient hospital stay for physical health reasons | £1,378 | Annual inflator to uprate the costs to 2021/2022 values (The Personal Social Services (PSS) Pay & Prices Index) | National Schedule of NHS Costs - Year 2020-21, Non Elective Inpatients (trim point floor of five days -https://www.england.nhs.uk/wp-content/uploads/2020/11/21-22NT_Annex-B-Guidance-on-currencies.pdf |
| Inpatient hospital stay for mental health reasons | £632 | Annual inflator to uprate the costs to 2021/2022 values (The Personal Social Services (PSS) Pay & Prices Index) | National Schedule of NHS Costs - Year 2020-21 - NHS trusts and NHS foundation trusts, PLICS Mental Health Provider Spells |
| **Outpatient contacts** | | | |
| General medical outpatient appointment | £235 | Outpatient attendances; Weighted average of all outpatient attendances | PSSRU 2022 |
| Day patient procedure/test | £229 | Annual inflator to uprate the costs to 2021/2022 values (The Personal Social Services (PSS) Pay & Prices Index) | National Schedule of NHS Costs - Year 2020-21, Outpatient Procedures |
| Hospital Memory clinic | £191 | Annual inflator to uprate the costs to 2021/2022 values (The Personal Social Services (PSS) Pay & Prices Index) | National Schedule of NHS Costs - Year 2020-21, PLICS Mental Health Care Contacts, Care Cluster 19 - Cognitive Impairment or Dementia Complicated (Moderate Need) |
| Psychiatric outpatient appointment | £684 | Annual inflator to uprate the costs to 2021/2022 values (The Personal Social Services (PSS) Pay & Prices Index) | National Schedule of NHS Costs - Year 2020-21, Total Outpatient Attendance, OLD AGE PSYCHIATRY SERVICE |
| **Community Group Support** | | | |
| Memory Group | £41 | Per client attendance; Private and voluntary sector day care for adults requiring mental health support (age 18-64) | PSSRU 2022 |
| Support Group provided for people with Dementia | £41 | Per client attendance; Private and voluntary sector day care for adults requiring mental health support (age 18-64) | PSSRU 2022 |
| Day centre | £58 | Per client attendance; Local authority own-provision social services day care for adults requiring mental health support (age 18-64) | PSSRU 2022 |
| State funded help for:   - Maintenance work and odd jobs - Cleaning the house, ironing and cooking - Personal Care | £25 | Per hour; Support and outreach worker | PSSRU 2022 |
| Family/ friends off work | £14.77 | Median hourly earnings of a carer in paid employment (year 2022) | https://www.ons.gov.uk/ |

GP General Practitioner; CMHT Community Mental Health Team; PSSRU Personal Social Services Research Unit

**Table S2: Baseline characteristics of participants randomised to adapted PATH and TAU**

|  | **adapted PATH** | **TAU** |
| --- | --- | --- |
| **Demographics** | | |
| N | 168 | 168 |
| Age, Median [IQR] (Range) | 78 [73,83] (54,97) | 76 [71,83.25] (52,94) |
| Gender (male), n (%) | 66 (39.3%) | 65 (38.7%) |
| **Ethnicity** | | |
| White British | 148 (88.1%) | 148 (88.1%) |
| White Irish | 2 (1.2%) | 5 (3.0%) |
| White Other | 6 (3.6%) | 7 (4.2%) |
| White & Black Caribbean | 1 (0.6%) | 1 (0.6%) |
| Other mixed background | 1 (0.6%) | 1 (0.6%) |
| Indian | 2 (1.2%) | 0 (0%) |
| Caribbean | 1 (0.6%) | 2 (1.2%) |
| African | 1 (0.6%) | 0 (0%) |
| Black Other | 1 (0.6%) | 0 (0%) |
| White & Asian | 0 (0%) | 1 (0.6%) |
| Other Asian background | 0 (0%) | 2 (1.2%) |
| Other | 5 (3.0%) | 1 (0.6%) |
| **Education** | | |
| Higher degree | 8 (4.8%) | 12 (7.1%) |
| Degree | 20 (11.9%) | 24 (14.3%) |
| A level (or equivalent) | 16 (9.5%) | 10 (6.0%) |
| HNC/HND (or equivalent) | 18 (10.7%) | 15 (8.9%) |
| NVQ (or equivalent) | 18 (10.7%) | 12 (7.1%) |
| GCSE (or equivalent) | 29 (17.3%) | 28 (16.7%) |
| School Leaving Certificate | 18 (10.7%) | 26 (15.5%) |
| No formal qualifications | 41 (24.4%) | 41 (24.4%) |
| **Marital status** | | |
| Married | 105 (62.5%) | 110 (65.5%) |
| Divorced | 14 (8.3%) | 7 (4.2%) |
| Single | 3 (1.8%) | 3 (1.8%) |
| Cohabiting | 6 (3.6%) | 5 (3.0%) |
| Widowed | 40 (23.8%) | 39 (23.2%) |
| Other | 0 (0%) | 4 (2.4%) |
| **Baseline antidepressant use** | | |
| Antidepressant prescription | 94 (56.0%) | 93 (55.4%) |
| **Score at baseline** | | |
| CSDD at baseline | 13 (3.8) | 12.8 (3.8) |
| Adjusted sMMSE at baseline | 21.9 (4.52) | 21.2 (4.68) |

IQR interquartile range; HNC/HND Higher National Certificates/Higher National Diplomas; NVQ National Vocational Qualification; GCSE General Certificate of Secondary Education; CSDD Cornell Score for Depression in Dementia; sMMSE Standardized Mini-Mental State Examination

**Figure S1: CONSORT flow diagram for the PATHFINDER trial**


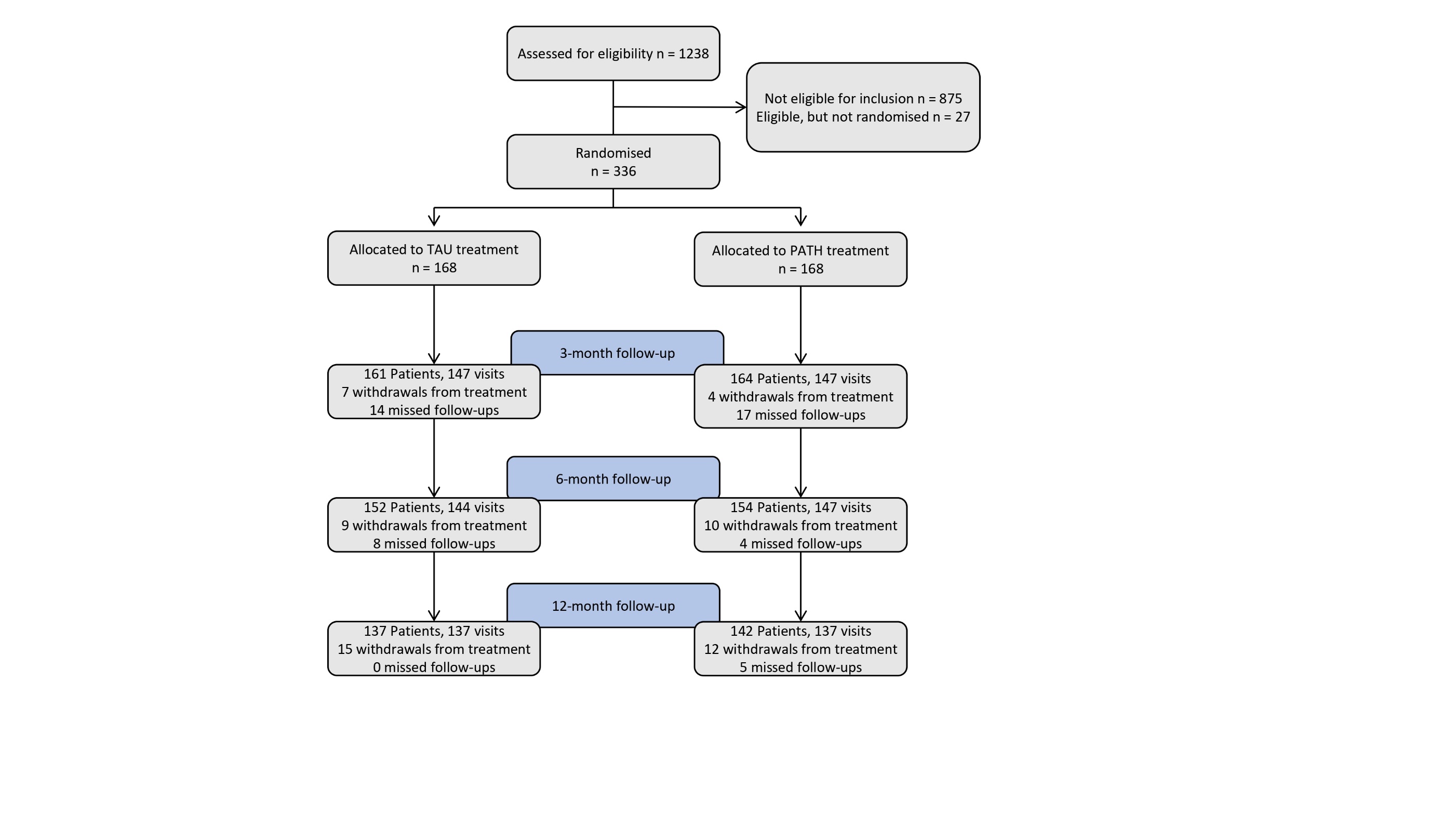


**Table S3. Baseline-adjusted mean difference in health service utilisation per participant** **over 12 months**

|  | **adapted PATH** | | **TAU** | |  | |
| --- | --- | --- | --- | --- | --- | --- |
|  | **N (n)** | **Mean (SD)** | **N (n)** | **Mean (SD)** | **Adjusted mean difference (95% CI)*** | **P value** |
| General practitioner (contacts) | 151 (90) | 3.72 (3.41) | 146 (93) | 4.16 (3.74) | -0.25 (-0.97 to 0.46) | 0.482 |
| Practice nurse (contacts) | 151 (53) | 3.21 (8.09) | 146 (59) | 2.64 (2.68) | 0.07 (-0.81 to 0.95) | 0.882 |
| Community/district nurse (contacts) | 151 (17) | 10.71 (20.22) | 147 (22) | 6.64 (10.21) | 0.34 (-1.02 to 1.71) | 0.622 |
| Memory Service/CMHT Doctor (contacts) | 151 (47) | 2.55 (3.05) | 147 (46) | 2.07 (1.98) | 0.14 (-0.27 to 0.55) | 0.510 |
| Memory Service /CMHT Occupational Therapist (contacts) | 151 (57) | 4.44 (5.46) | 147 (57) | 4.04 (4.77) | 0.14 (-0.69 to 0.96) | 0.748 |
| Memory Service /CMHT Social worker (contacts) | 151 (14) | 3.57 (4.16) | 147 (9) | 3.44 (3.64) | 0.12 (-0.18 to 0.43) | 0.429 |
| Memory Service /CMHT Psychologist (contacts) | 152 (17) | 4.71 (7.41) | 147 (19) | 3.68 (3.84) | 0.16 (-0.34 to 0.65) | 0.538 |
| Memory Service /CMHT Assistant Practitioner (contacts) | 151 (13) | 5.38 (5.72) | 147 (17) | 4.65 (5.65) | -0.18 (-0.65 to 0.29) | 0.454 |
| Other community Practitioner (contacts) | 151 (20) | 2.05 (1.19) | 147 (24) | 5.33 (8.06) | **-0.63 (-1.24 to -0.02)** | **0.041** |
| **All Primary care services (contacts)** | 152 (127) | 10.24 (12.69) | 147 (133) | 9.94 (10.04) | -0.61 (-2.95 to 1.72) | 0.606 |
| Psychological therapy (contacts) | 151 (15) | 4.27 (3.15) | 147 (13) | 6.46 (5.41) | 0.06 (-0.29 to 0.42) | 0.736 |
| Emergency services (contacts) | 151 (48) | 2.52 (1.79) | 147 (54) | 2.89 (3.78) | -0.26 (-0.76 to 0.23) | 0.301 |
| Overnight Inpatient stay (nights) | 152 (17) | 13.53 (15.91) | 147 (25) | 17.36 (17.86) | -1.51 (-3.40 to 0.38) | 0.118 |
| Outpatient services (contacts) | 151 (78) | 3.10 (2.66) | 147 (83) | 3.42 (5.84) | -0.37 (-1.18 to 0.44) | 0.370 |
| Community Group Support (contacts) | 151 (35) | 9.89 (10.54) | 147 (42) | 15.1 (20.65) | -2.05 (-4.18 to 0.08) | 0.060 |
| Medication (prescriptions) | 132 (132) | 5.33 (3.55) | 133 (131) | 5.14 (3.62) | 0.02 (-0.77 to 0.82) | 0.952 |
| State funded help (contacts) | 168 (114) | 6.43 (4.14) | 168 (109) | 6.79 (4.08) | -0.14 (-0.96 to 0.68) | 0.733 |
| Privately funded help (contacts) | 168 (114) | 5.08 (3.61) | 168 (109) | 5.39 (3.52) | -0.09 (-0.77 to 0.59) | 0.794 |
| Unpaid help (contacts) | 168 (73) | 2.41 (1.15) | 168 (69) | 2.29 (1.15) | 0.07 (-0.21 to 0.36) | 0.606 |

*Baseline-adjusted differences calculated using bias-corrected and accelerated bootstrapped regressions

CMHT Community Mental Health Team; CI confidence interval; SD standard deviation

N number of participants in each arm; n number of participants with responses to resource use questionnaires

**Table S4. Baseline-adjusted mean difference in health service costs (£) per participant over 12 months**

|  | **adapted PATH** | | **TAU** | |  |  |
| --- | --- | --- | --- | --- | --- | --- |
|  | **N (n)** | **Mean (SD)** | **N (n)** | **Mean (SD)** | **Adjusted mean difference (95% CI)*** | **P value** |
| General practitioner | 151 (90) | £146 (£253) | 146 (93) | £144 (£327) | -£6 (-£60 to £49) | 0.836 |
| Practice nurse | 151 (53) | £54 (£179) | 146 (59) | £37 (£53) | £4 (-£14 to £23) | 0.651 |
| Community/district nurse | 151 (17) | £707 (£1,409) | 147 (22) | £379 (£700) | £33 (-£60 to £126) | 0.488 |
| Memory Service/CMHT Doctor (contacts) | 151 (47) | £102 (£112) | 147 (46) | £89 (£97) | £3 (-£13 to £20) | 0.714 |
| Memory Service /CMHT Occupational Therapist (contacts) | 151 (57) | £84 (£135) | 147 (57) | £75 (£81) | £4 (-£13 to £22) | 0.642 |
| Memory Service /CMHT Social worker (contacts) | 151 (14) | £48 (£46) | 147 (9) | £60 (£66) | £1 (-£3 to £5) | 0.697 |
| Memory Service /CMHT Psychologist (contacts) | 152 (10) | £146 (£148) | 147 (10) | £133 (£47) | £0.5 (-£9 to £10) | 0.924 |
| Memory Service /CMHT Assistant Practitioner (contacts) | 151 (13) | £57 (£61) | 147 (17) | £48 (£67) | -£2 (-£7 to £4) | 0.542 |
| Other community Practitioner (contacts) | 151 (20) | £37 (£37) | 147 (24) | £200 (£515) | -£29 (-£63 to £6) | 0.108 |
| Psychological therapy (contacts) | 151 (15) | £168 (£128) | 147 (13) | £215 (£189) | £5 (-£8 to £18) | 0.437 |
| Emergency services (contacts) | 151 (48) | £385 (£326) | 147 (54) | £427 (£784) | -£35 (-£128 to £57) | 0.452 |
| Overnight Inpatient stay (nights) | 152 (17) | £11,063 (£14,008) | 147 (25) | £15,968 (£18,204) | -£1,542 (-£3,317 to £233) | 0.089 |
| Outpatient services (contacts) | 151 (78) | £715 (£616) | 147 (83) | £789 (£1,367) | -£84 (-£273 to £104) | 0.381 |
| Community Group Support (contacts) | 151 (35) | £405 (£432) | 147 (42) | £619 (£846) | -£84 (-£171 to £3) | 0.060 |
| Medication | 131 (131) | £149 (£329) | 130 (129) | £71 (£124) | £46 (-£2 to £94) | 0.060 |
| Adaptations | 151 (38) | £3,191 (£8,056) | 147 (37) | £3,672 (£7,351) | -£70 (-£989 to £848) | 0.880 |
| State funded help | 168 (23) | £7,288 (£12,373) | 168 (26) | £4,460 (£5,552) | £311 (-£572 to £1,195) | 0.490 |
| Privately funded help | 168 (75) | £8,449 (£28,772) | 168 (68) | £19,047 (£56,232) | -£550 (-£3,983 to £2,883) | 0.753 |
| Unpaid help | 168 (42) | £1,218 (£1,648) | 168 (37) | £754 (£1,217) | £163 (-£11 to £336) | 0.066 |
| Family/friend help | 168 (31) | £2,580 (£5,061) | 168 (21) | £1,058 (£1,251) | £318 (-£28 to £667) | 0.072 |

*Baseline-adjusted differences calculated using bias-corrected and accelerated bootstrapped regressions

CMHT Community Mental Health Team; CI confidence interval; SD standard deviation

N number of participants in each arm; n number of participants with responses to resource use questionnaires

**Table S5. Mean utility scores at baseline and each follow-up point generated from the EQ-5D-5L and DEMQOL/DEMQOL-Proxy and baseline- adjusted mean QALYs over 12 months**

|  | **adapted PATH** | | **TAU** | |  |  |
| --- | --- | --- | --- | --- | --- | --- |
|  | **N (n)** | **Mean (SD)** | **N (n)** | **Mean (SD)** | **Adjusted mean difference (95% CI)*** | **P value** |
| **EQ-5D-5L** | | | | | | |
| Baseline | 164 (160) | 0.680 (0.215) | 166 (161) | 0.691 (0.223) |  |  |
| 3 months | 135 (132) | 0.700 (0.239) | 138 (133) | 0.722 (0.210) |  |  |
| 6 months | 127 (126) | 0.707 (0.236) | 122 (116) | 0.710 (0.224) |  |  |
| 12 months | 109 (108) | 0.713 (0.223) | 106 (100) | 0.728 (0.224) |  |  |
| QALYs | 99 (99) | 0.692 (0.219) | 99 (97) | 0.702 (0.190) | **0.047 (0.011 to 0.083)** | **0.011** |
| **DEMQOL patient** | | | | | | |
| Baseline | 162 (162) | 0.781 (0.144) | 160 (160) | 0.797 (0.125) |  |  |
| 3 months | 120 (120) | 0.806 (0.137) | 130 (130) | 0.801 (0.134) |  |  |
| 6 months | 123 (123) | 0.814 (0.123) | 116 (116) | 0.806 (0.143) |  |  |
| 12 months | 94 (94) | 0.815 (0.140) | 90 (90) | 0.831 (0.116) |  |  |
| QALYs | 82 (82) | 0.820 (0.105) | 81 (81) | 0.821 (0.094) | 0.009 (-0.015 to 0.033) | 0.462 |
| **DEMQOL-Proxy** | | | | | | |
| Baseline | 163 (163) | 0.648 (0.128) | 164 (164) | 0.655 (0.132) |  |  |
| 3 months | 137 (137) | 0.682 (0.125) | 139 (139) | 0.652 (0.133) |  |  |
| 6 months | 136 (136) | 0.674 (0.132) | 127 (127) | 0.672 (0.117) |  |  |
| 12 months | 125 (125) | 0.667 (0.113) | 112 (112) | 0.656 (0.126) |  |  |
| QALYs | 106 (106) | 0.676 (0.090) | 105 (105) | 0.663 (0.087) | 0.012 (-0.007 to 0.030) | 0.219 |

*Baseline-adjusted differences calculated using bias-corrected and accelerated bootstrapped regressions

CI confidence interval; SD standard deviation

N number of participants in each arm; n number of participants with responses to health-related quality-of-life questionnaires

**Fig S2. Cost-effectiveness plane and cost-effectiveness acceptability curves of adapted PATH compared to TAU based on sensitivity analysis assessing the impact of the adapted PATH intervention delivery costs only, excluding training and consumables costs (NHS/PSS perspective) at 12 months**

**Fig S3. Cost-effectiveness acceptability curves of adapted PATH compared to TAU based on sensitivity analyses assessing the impact of COVID-19 restrictions on the results (NHS/PSS and societal perspectives)**  **at 12 months**

**Fig S4 Cost effectiveness planes of adapted PATH compared to TAU based on sensitivity analysis assuming three MNAR scenarios** **(NHS/PSS perspective)**

**Fig S5 Cost-effectiveness acceptability curves of adapted PATH compared to TAU based on sensitivity analysis assuming three MNAR scenarios (NHS/PSS perspective)**
